# Feeding Type at 6–12 Months and Early-Life Iron-Related Hematologic Changes in Infants: A Longitudinal Birth Cohort Study

**DOI:** 10.3390/nu18132197

**Published:** 2026-07-06

**Authors:** Jiyoung Jeong, Minyoung Jung, Jeongmin Song, Sukyung Kim, Joonghyun Ahn, Mijeong Kwon, Mi Jin Kim, Yoon Jung Yang, Jihyun Kim, Kangmo Ahn

**Affiliations:** 1 Department of Clinical Nutrition, Graduate of Future Strategy Convergence, Dongduk Women’s University, Seoul 02748, Republic of Korea; 2 Department of Pediatrics, Samsung Medical Center, Sungkyunkwan University School of Medicine, Seoul 06351, Republic of Korea; my7834my@gmail.com (M.J.); jumi1211@naver.com (J.S.); mijin1217.kim@samsung.com (M.J.K.);; 3 Biomedical Statistics Center, Data Science Research Institute, Samsung Medical Center, Seoul 06351, Republic of Korea; 4 Department of Food and Nutrition, Dongduk Women’s University, Seoul 02748, Republic of Korea; 5 Department of Health Sciences and Technology, Samsung Advanced Institute for Health Sciences & Technology, Seoul 06351, Republic of Korea

**Keywords:** breastfeeding, formula feeding, iron status, infant growth, birth cohort study

## Abstract

Background/Objectives: The World Health Organization recommends continued breastfeeding for up to 2 years of age. However, because breast milk contains less iron than formula milk, prolonged exclusive breastfeeding may increase the risk of iron deficiency anemia. This study aimed to compare iron status and growth outcomes at 12 and 36 months based on feeding methods during the 6–12-month period. Methods: In this prospective birth cohort study, we followed 126 children from birth to 36 months. Participants were categorized into breastfeeding (BF, *n* = 21), formula feeding (FF, *n* = 62), and mixed feeding (MF, *n* = 43) groups according to milk feeding over 6–12 months of age. Anthropometric measurements and iron parameters were compared among groups at 12 and 36 months. Results: At 12 months, the BF group had significantly lower weight and height compared to the MF group. Hemoglobin, mean corpuscular hemoglobin, and mean corpuscular hemoglobin concentration were significantly lower in the BF group than in the FF and MF groups, while red cell distribution width was significantly higher in the BF group. At 36 months, no differences in anthropometrics or iron parameters were observed among the groups. Conclusions: Infants breastfed without formula from 6 to 12 months showed lower iron-related hematologic parameters at 12 months, which largely resolved by 36 months. These findings highlight late infancy as a potentially vulnerable period for insufficient iron intake and support the importance of adequate iron supplementation and timely introduction of iron-rich complementary foods during this critical developmental stage.

## 1. Introduction

Iron deficiency remains a significant global health issue, particularly among infants during critical periods of growth and development [1,2]. Adequate iron stores in the first year of life are essential to support erythropoiesis and neurocognitive development [3]. In early infancy, most healthy, full-term infants rely on iron reserves accumulated prenatally, along with small amounts of highly bioavailable iron from breast milk [4]. This iron supply generally meets physiological needs up to around four to six months of age [5].

Both the American Academy of Pediatrics (AAP) and the World Health Organization (WHO) recommend exclusive breastfeeding for around the first 6 months of life [6,7]. A Cochrane Systematic Review also supports exclusive breastfeeding for the first six months of life in both developing and developed-country settings [8]. Despite increasing iron requirements as infants grow, the WHO and other health authorities encourage continued breastfeeding for up to 24 months [7]. Breast milk remains a vital source of macronutrients and micronutrients such as protein, vitamin A, calcium, and riboflavin, particularly in the second year of life, especially in resource-limited settings where complementary foods may be insufficient [9]. In addition, breastfeeding continues to offer immune protection, and plays a critical role in maternal health by promoting birth spacing and reducing the risks of certain cancers, type 2 diabetes, and cardiovascular disease [10,11,12,13].

However, prolonged exclusive breastfeeding without adequate iron supplementation may increase the risk of iron deficiency anemia (IDA) as infants’ iron requirements increase [14,15]. Around 6 months of age, full-term infants require additional dietary iron, with standard dietary guidelines recommending the introduction of iron-rich solid foods as the primary source [15]. For infants not yet consuming such foods, the WHO recommends iron supplementation from four to six months [16]. Nevertheless, a meta-analysis of daily iron supplementation in healthy exclusively breastfed infants showed no significant reduction in iron deficiency and indicated possible slow growth during breastfeeding [17].

Given these challenges and inconsistencies in current recommendations, further research is needed to understand the effects of different feeding practices on infant health outcomes. Therefore, this study aimed to evaluate the short- and long-term effects of milk feeding practices during 6–12 months on anthropometric measures and iron-related hematologic parameters in a prospective birth cohort.

## 2. Materials and Methods

### 2.1. Study Population and Design

This study is a secondary analysis of a prospective birth cohort originally established at Samsung Medical Center (Seoul, Republic of Korea) between 2017 and 2020 to investigate early-life environmental factors and allergic diseases. We screened 243 pregnant women at 28 weeks of gestation or later, of whom 82 failed to meet the eligibility criteria. Participants were categorized into two groups: the high-risk group (at least one parent diagnosed with asthma, allergic rhinitis, or atopic dermatitis with allergen sensitization, or at least one sibling with atopic dermatitis) and the control group (no family history of allergic diseases and negative skin prick test results for all allergens in the mother). Of the 161 mothers (162 infants, including one set of twins) who passed the screening, 25 were excluded due to study withdrawal or revocation of consent. Consequently, 137 participants were ultimately enrolled in the study [18]. At enrollment, parents completed a questionnaire on basic demographic information. Infants were regularly followed at 2, 6, 12, and 36 months of age. Out of 137 participants, 126 who provided complete data on milk feeding practices for 6–12 months were included in the analysis. This study was approved by the Institutional Review Board of Samsung Medical Center (IRB No. SMC–2016–12-111), and written informed consent was obtained from all parents before participation in this study. The study protocol was registered on the World Health Organization International Clinical Trials Registry Platform (registration no. KCT0007979).

### 2.2. Collection of Demographic Data

Information on sex, gestational age, and birth mode was obtained from the parental questionnaire. Birth weight was measured to the nearest 0.1 kg. We defined preterm infants as those born before 37 weeks of gestation. A family history of allergic diseases was established when 1 of 2 criteria was met: at least one parent had both a positive skin test response and a history of asthma or allergic rhinitis, or at least one parent or sibling had physician-diagnosed atopic dermatitis.

### 2.3. Evaluation of Food Intake Status

Between 6 and 12 months of age, all infants received complementary foods and were categorized into three groups based on the type of milk consumed: the breastfeeding (BF) group (*n* = 21; breast milk only, without formula), the formula feeding (FF) group (*n* = 62; formula only, without breast milk), and the mixed feeding (MF) group (*n* = 43; both breast milk and formula). Although specific infant formula brands were not individually recorded, all formulas distributed in South Korea are strictly regulated by the Ministry of Food and Drug Safety (MFDS) [19], which mandates a standardized iron content of 0.5–2.0 mg per 100 kcal. According to recent market share data [20], the leading brands—including Aptamil (Nutricia), Absolute (Maeil Dairy), and Imperial Dream (Namyang Dairy)—consistently maintain iron levels of approximately 1.0–1.2 mg per 100 kcal. Thus, the variation in iron intake from different formula brands was considered negligible.

Parents responded to a food frequency questionnaire (FFQ) at 12 months regarding their infant’s consumption of various foods over the past month. The FFQ consists of questions on 33 foods or food groups. The foods were categorized into 10 groups: cereals and starch (rice, potato, wheat products), egg, meat, fish, bean, vegetable, fruit, milk and dairy (excluding formula milk), and nuts and oil. Parents were asked about their children’s iron supplement intake and the introduction of complementary foods through a questionnaire.

### 2.4. Anthropometric and Iron Parameter Evaluation

Anthropometric measurements were performed at 2, 6, 12, and 36 months of age by trained research staff following standardized protocols. At 2, 6, and 12 months, recumbent length and weight were measured simultaneously using an infantometer with an integrated digital scale (JENIX, Dong Sahn Jenix Co., Ltd., Seoul, Republic of Korea), with the child wearing only a clean disposable diaper. Recumbent length was recorded to the nearest 0.1 cm, and weight to the nearest 0.01 kg. At 36 months, standing height was measured to the nearest 0.1 cm using a portable stadiometer (Dong Sahn Jenix Co., Ltd., Seoul, Republic of Korea), with the child barefoot, and the head positioned in the Frankfurt plane. Weight was measured to the nearest 0.1 kg using a digital scale (Dong Sahn Jenix Co., Ltd., Seoul, Republic of Korea), with the child wearing light indoor clothing; 0.2 kg was subtracted from the measured weight to account for clothing weight. Head circumference was measured at each visit using a non-stretchable measuring tape placed just above the eyebrows and around the occipital prominence, and recorded to the nearest 0.1 cm. Infant growth Z-scores were calculated based on the WHO Child Growth Standards using the Lambda–Mu–Sigma (LMS) method [21].

Hematological parameters, including hemoglobin (Hb), hematocrit (Hct), mean corpuscular volume (MCV), mean corpuscular hemoglobin (MCH), mean corpuscular hemoglobin concentration (MCHC), and red cell distribution width (RDW), were analyzed using an automated hematology analyzer (Sysmex XN–series, Sysmex Corporation, Kobe, Japan). We defined a binary outcome for iron deficiency parameter standard cutoffs based on age as follows: Hb < 10.5 g/dL, Hct < 33%, MCV < 70 fL, MCH < 23 pg, MCHC < 30%, and RDW ≥ 15 [20].

### 2.5. Statistical Analysis

Data were analyzed using SAS 9.4 (SAS Institute Inc., Cary, NC, USA) and R version 4.5.1 (R Foundation for Statistical Computing, Vienna, Austria). Baseline characteristics are summarized with descriptive statistics. Continuous variables, including hematologic parameters and daily food group intake, are expressed as medians with interquartile range (IQR), and categorical variables are presented as counts and percentages. Normality was verified for the BF, FF, and MF groups using the Shapiro–Wilk test. As multiple variables deviated from a normal distribution, intergroup differences were analyzed via the Kruskal–Wallis test. Fisher’s exact test was applied to categorical variables due to the high proportion of cells with expected counts below 5. For post hoc comparisons, the Dwass–Steel–Critchlow–Fligner (DSCF) procedure was used. To compare food intake at 12 months, and anthropometric and hematologic parameters at 36 months among the BF, FF, and MF groups, rank-based analysis of covariance (Rank-based ANCOVA) was employed, controlling for covariates. Subsequent post hoc testing was conducted via pairwise comparisons of the ranks. To evaluate longitudinal changes in anthropometric parameters (weight, height, and head circumference) according to feeding, we applied generalized estimating equations (GEEs) with an autoregressive (AR(1)) working correlation structure to account for within-subject correlation over time. Follow-up time was modeled as a continuous variable using natural cubic splines (with 3 degrees of freedom) to flexibly capture nonlinear growth trajectories. Feeding type was treated as a three-level categorical predictor, and sex and prematurity were adjusted as covariates. *p* values < 0.05 were considered statistically significant.

## 3. Results

### 3.1. Clinical Characteristics of the Study Subjects

Demographic characteristics of the subjects at birth are described in Table 1. No differences were found among feeding groups in terms of birth weight, height, head circumference, sex, gestational age, prematurity, and delivery mode. The BF group showed a significantly lower weight-for-age Z-score compared to the FF group (*p* = 0.012). The height-for-age Z-score in the BF group was significantly lower than that of both the FF and MF groups (*p* = 0.002).

### 3.2. Feeding Practice Assessment of Study Participants Aged 12 Months

The timing of complementary food introduction showed significant variation across the feeding groups (*p* = 0.011) (Table 2). The proportion of infants who began to eat complementary foods at or after 6 months was significantly higher in the BF group (42.86%) than in the FF (11.29%) and MF (28.57%) groups. Among the study participants taking iron supplements, the proportion in the BF group (43.75%) was significantly higher than that in the FF (8.16%) and MF (12.9%) groups (*p* = 0.003).

Table 2 presents the daily food group intake at 12 months according to feeding group from 6 to 12 months. Dairy product consumption was significantly higher in the FF group at 6.0 times/day, compared to the BF group at 3.9 times/day (*p* = 0.033). However, there was no statistically significant difference in the frequency of intake of food types among the feeding groups, so cereal and starch, egg, meat, fish, bean, vegetable, fruit, nut, and oil were not significantly associated with the feeding group (all *p* > 0.05).

### 3.3. Longitudinal Changes in Anthropometric Parameters by Feeding Type

**Figure 1:**
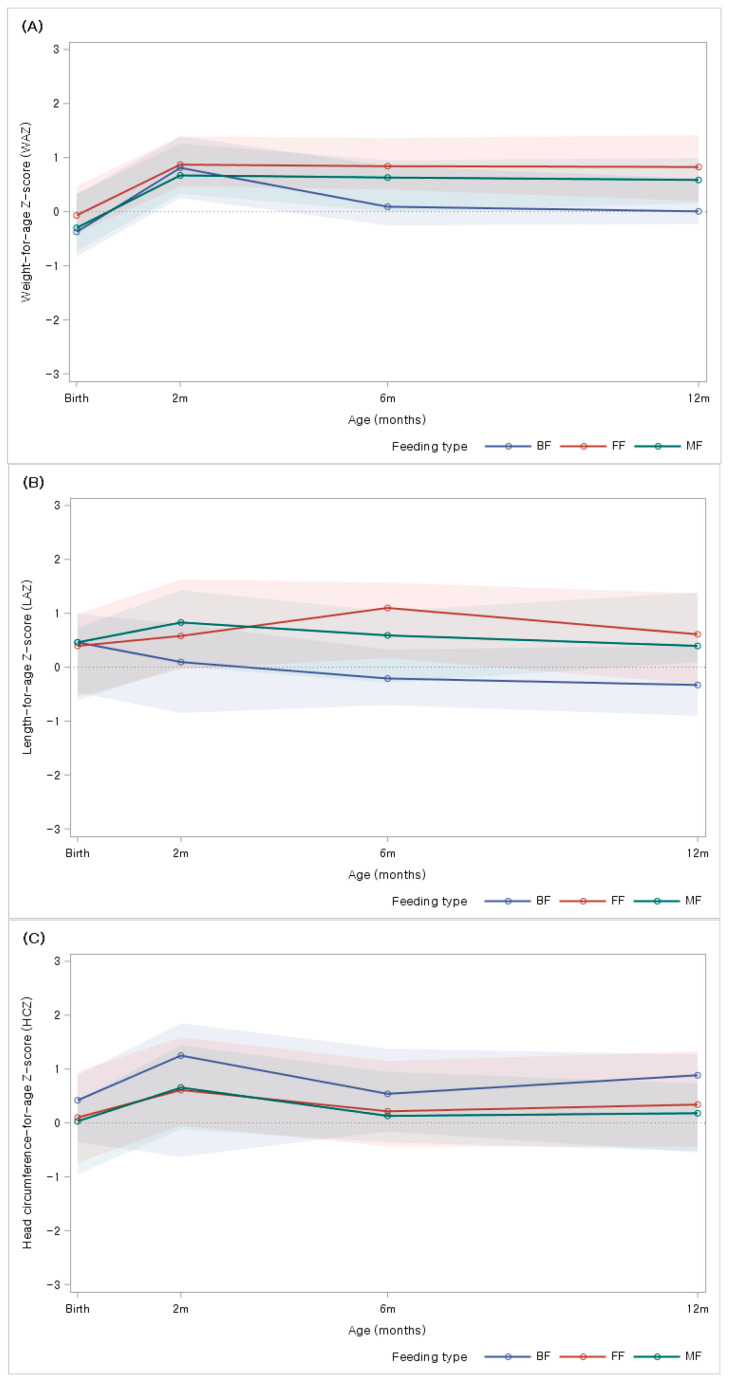
Trends in growth outcomes by feeding type from birth to 12 months: (**A**) weight, (**B**) height, and (**C**) head circumference. Lines represent the median and shaded areas represent interquartile range (IQR).

Significant differences in weight Z-scores emerged at 6 months (Figure 1A), where the BF group exhibited lower weight Z-scores compared to both the FF group (Estimate = −0.51, *p* = 0.004) and the MF group (Estimate = −0.56, *p* = 0.006, Table S1). By 12 months, the BF group continued to show a significantly lower weight Z-score specifically compared to the MF group (Estimate = −0.64, *p* = 0.006). Regarding height Z score, the BF group at 2 months was significantly shorter than the MF group (Estimate= −0.62, *p* = 0.008, Table S2). At 12 months, the BF group remained significantly shorter than the MF group (Estimate = −0.76, *p* = 0.001; Table S2) (Figure 1B).

In contrast to weight and height trajectories, head circumference Z-score (HCZ) did not show any statistically significant differences between the feeding groups at any follow-up time point (Figure 1C and Table S3).

### 3.4. Iron-Related Hematologic Parameters at 12 Months

There were significant differences in hematological parameters at 12 months among the feeding groups. Median (IQR) Hb levels were significantly lower in the BF group (11.2 [10.9–12.4] g/dL) compared to the FF (12.6 [12.2–13.1] g/dL) and MF groups (12.2 [11.8–12.8] g/dL) (*p* < 0.001) (Table 3). Both median (IQR) MCH and MCHC were lower in the BF group than in the FF and MF groups (both for *p* < 0.001). RDW was higher in the BF group (13.2 [12.5–15.0] %) compared to the FF group (12.7 [12.1–13.2] %) and MF group (12.7 [12.1–13.2]) with statistical significance (*p* = 0.006). Hct and MCV at 12 months did not show statistically significant differences among the groups.

### 3.5. Anthropometric and Iron Parameters at 36 Months

Anthropometric and iron parameters at 36 months did not differ significantly among the feeding groups (Table 4), in contrast to the significant differences observed at 12 months.

## 4. Discussion

This longitudinal birth cohort study examined the association between milk feeding practices and growth and iron-related hematologic parameters up to 36 months. Infants breastfed without formula during 6–12 months showed differences in growth trajectories, with lower anthropometric measures at 12 months when assessed using standardized metrics, and a higher prevalence of anemia with elevated RDW values, suggesting a pattern compatible with iron-deficient erythropoiesis. By 36 months, these early differences had largely attenuated, with no significant anthropometric or hematologic parameters when assessed using absolute values. Even if these disparities appear to be attenuated by 36 months, further research is warranted to determine the long-term consequences of inadequate iron intake in late infancy and to develop feeding guidelines that support breastfeeding while ensuring sufficient iron intake.

Importantly, these findings should not be interpreted as indicating abnormal growth in breastfed infants but rather as reflecting differences in growth trajectories across feeding types. Standardized growth references were applied to allow for appropriate comparisons across groups. During the first six months, iron needs are met by prenatal iron stores and the small amount of highly bioavailable iron in breast milk. After this period, iron stores are depleted, and the demand for iron rises sharply due to rapid tissue growth [23]. Since breast milk contains a low amount of iron, exclusive breastfeeding beyond 6 months without iron supplementation increases the risk of anemia [24]. A birth cohort study indicated that exclusive breastfeeding for ≥6 months, as opposed to 3–5 months, was significantly associated with an increased risk of anemia in infants at 12 months of age, compared to exclusive breastfeeding for less than 3 months [14]. Another birth cohort study showed that infants exclusively breastfed for ≥6 months were more likely to develop anemia, compared to those exclusively breastfed for less than 4 months [25].

In our cohort, the BF group at 12 months exhibited lower Hb, MCH, and MCHC, and higher RDW, compared with the FF and MF groups, suggesting a pattern compatible with iron-deficient erythropoiesis. IDA during the first year of life has been associated with alterations in neurodevelopmental outcomes, including deficits in cognitive performance, motor skills, and socio-emotional behavior, which may persist beyond the period of deficiency, despite subsequent iron repletion [26]. In addition, longitudinal follow-up studies in cohorts assessed from preschool age through adolescence have demonstrated that children with IDA in infancy exhibit persistent deficits in executive function, spatial memory, and selective attention, as well as long-lasting neurophysiologic alterations in auditory brainstem response and visual evoked potentials [27,28]. These findings highlight the potential for early iron deficiency to induce enduring alterations in neural circuitry and functional outcomes. Notably, these differences were observed despite a significantly higher proportion of iron supplementation in the BF group compared with the other feeding groups, suggesting that supplementation alone, without attention to timing, dosage, formulation, and overall dietary patterns, may not have been sufficient in some infants to normalize hematologic indices during late infancy.

Nevertheless, breastfeeding remains the optimal source of infant nutrition, providing highly bioavailable nutrients and immune protection through immunoglobulins and bioactive factors, as well as fostering mother–infant bonding. The challenge lies in preserving these benefits while minimizing the risk of iron deficiency during late infancy. Strategies to prevent iron deficiency during late infancy address both dietary sources and optimal intervention timing. For infants, iron-rich complementary foods (such as red meat and iron-fortified cereals) should be introduced starting at 6 months of age, when prenatal iron stores are typically depleted [29,30]. In addition, the American Academy of Pediatrics recommends that exclusively or predominantly breastfed term infants receive oral iron supplementation (e.g., liquid drops) at a dose of 1 mg/kg per day, beginning at 4 months of age and continued until adequate iron-containing complementary foods are introduced [31,32]. In addition, although maternal dietary iron intake does not alter the iron concentration in breast milk [33], vitamin C, a major dietary enhancer of non-heme iron absorption, increases in breast milk [34]. Because the small amount of iron in breast milk is highly bioavailable, ensuring that lactating mothers maintain adequate vitamin C status through a balanced diet rich in fruits and vegetables may further enhance iron bioavailability and indirectly support optimal iron status in breastfed infants.

One limitation of this study is the relatively small sample size, which may affect the generalizability of the findings. In addition, we did not include direct biochemical measures of iron status, such as serum ferritin, transferrin saturation, or soluble transferrin receptor, and maternal iron status. Furthermore, this study lacks a precise quantification of daily iron intake from complementary foods and did not record the specific chemical formulations of the iron supplements used (e.g., ferrous sulfate, gluconate, or bisglycinate).

Although the higher prevalence of anemia accompanied by elevated RDW values in the breastfeeding group suggests a possible increased risk of IDA, these findings should be interpreted with caution, as RDW is an indirect marker and can be influenced by other conditions, such as vitamin B_12_ or folate deficiency, chronic disease anemia, or recovery from acute illness. The attenuation of differences by 36 months may reflect a combination of biological catch-up growth and changes in dietary intake during the later infancy and toddler period. However, the lack of detailed dietary data between 13 and 36 months limits our ability to disentangle the relative contributions of these factors. In addition, maternal factors that may influence infant growth, such as gestational diabetes or mid-parental height, were not systematically assessed in this study and could not be accounted for in the analysis. Another limitation of this study is the baseline difference in physical parameters at birth. We cannot entirely rule out the possibility that these baseline differences influenced the growth trajectories observed from birth to 12 months. Although baseline anthropometric differences may have influenced subsequent growth trajectories, the hematologic differences observed at 12 months remained significant after covariate adjustment. Nevertheless, a key strength of this study is its longitudinal design, allowing repeated measurement of growth and hematologic outcomes from birth to 36 months. This approach provides evidence of potential nutritional vulnerabilities during the 6–12 months window, underscoring the need for targeted dietary interventions during this critical developmental stage.

## 5. Conclusions

Breastfeeding without formula from 6 to 12 months was associated with transient differences in hematologic parameters suggestive of iron-deficient erythropoiesis at 12 months, despite higher reported iron supplementation. Although these differences were largely attenuated by 36 months, late infancy may represent a potentially vulnerable period for insufficient iron intake among some breastfed infants. Strategies including timely introduction of iron-rich complementary foods, adequate iron supplementation, and regular monitoring may help support optimal iron status while preserving the established benefits of breastfeeding.

## Figures and Tables

**Table 1:** Clinical characteristics of study participants.

Characteristics	BF	FF	MF	*p* Value ^1^
	(*n* = 21)	(*n* = 62)	(*n* = 43)	
Birth weight (kg)	3.08 (2.95–3.49)	3.30 (3.13–3.53)	3.19 (2.91–3.43)	0.085
Weight-for-age Z-score	0.05 (−0.14–0.74) ^a2^	0.83 (0.20–1.42) ^b^	0.59 (0.15–1.00) ^ab^	0.012
Birth height (cm)	50.00 (49.00–51.00)	50.50 (48.75–51.00)	50.00 (48.00–51.00)	0.700
Height-for-age Z-score	−0.04 (−0.47–0.46) ^a^	0.91 (−0.02–1.67) ^b^	0.68 (0.37–1.69) ^b^	0.002
Birth head circumference (cm)	35.00 (34.00–35.00)	34.50 (33.00–35.50)	34.00 (33.25–34.50)	0.479
Head Circumference-for-age Z-score	0.81 (−0.51–1.27)	0.34 (−0.44–1.33)	0.18 (−0.52–0.73)	0.493
Sex				0.271
Male	9 (42.86)	39 (62.90)	27 (62.79)	
Female	12 (57.14)	23 (37.10)	16 (37.21)	
Gestational age at birth (wk)	40.00 (39.40–40.01)	39.15 (38.40–39.60)	39.40 (38.40–40.20)	0.548
Preterm				0.210
Yes	2 (9.52)	1 (1.61)	3 (6.98)	
No	19 (90.48)	61 (98.39)	40 (93.02)	
Birth mode				0.391
Vaginal delivery	13 (61.90)	38 (61.29)	21 (48.84)	
Cesarean section	8 (38.10)	24 (38.71)	22 (51.16)	
Family history of allergic disease				0.427
Yes	13 (61.90)	45 (72.58)	26 (60.47)	
No	8 (38.10)	17 (27.42)	17 (39.53)	

Abbreviation; BF, breastfeeding; FF, formula feeding; MF, mixed feeding. Values are presented as number (%) or median (IQR). ^1^ *p*-values obtained by Kruskal–Wallis test for continuous variables and Fisher’s exact test for categorical variables. ^2^ Post hoc testing was performed using the Dwass–Steel–Critchlow–Fligner (DSCF) method; medians within a row with different superscript letters were significantly different (*p* < 0.05).

**Table 2:** Feeding practice of study participants at 12 months.

	BF	FF	MF	*p* Value ^1^
	(*n* = 21)	(*n* = 62)	(*n* = 43)	
Introduction of complementary food				0.011
≥4 month	6 (28.57)	13 (20.97)	9 (21.43)	
≥5 month	6 (28.57)	42 (67.74)	21 (50.00)	
≥6 month	9 (42.86)	7 (11.29)	12 (28.57)	
Iron supplements ^2^				0.003
Yes	7 (43.75)	4 (8.16)	4 (12.90)	
No	9 (56.25)	45 (91.84)	27 (87.10)	
Food groups (frequency/day)				
Cereal and starch	3.12 (2.21–7.30)	3.76 (3.00–6.67)	3.25 (2.30–4.43)	0.342
Egg	0.54 (0.00–0.86)	0.33 (0.03–0.86)	0.50 (0.08–0.86)	0.478
Meat	1.00 (0.79–2.00)	1.50 (0.79–3.00)	2.00 (0.79–3.00)	0.670
Fish	0.21 (0.00–0.21)	0.21 (0.08–0.79)	0.21 (0.08–0.50)	0.103
Bean	0.50 (0.08–0.79)	0.25 (0.21–0.79)	0.21 (0.08–0.87)	0.885
Vegetable	7.63 (5.43–8.36)	8.15 (5.64–8.46)	7.50 (5.91–9.08)	0.865
Fruit	1.03 (1.00–1.21)	1.00 (0.71–2.00)	1.00 (0.79–1.03)	0.576
Dairy products	3.90 (0.00–6.00) ^a3^	6.00 (4.07–7.00) ^b^	5.00 (3.00–6.43) ^ab^	0.033
Nut	0.00 (0.00–0.03)	0.00 (0.00–0.00)	0.00 (0.00–0.00)	0.254
Oil	0.43 (0.21–0.64)	0.19 (0.00–0.89)	0.07 (0.03–1.00)	0.260

Abbreviation; BF, breastfeeding; FF, formula feeding; MF, mixed feeding. Values are presented as number (%) or median (IQR). ^1^ *p*-values obtained by rank-based analysis of covariance (rank-based ANCOVA) for continuous variables after adjusting for sex, birth weight, and premature infant, and Fisher’s exact test for categorical variables. ^2^ The questionnaire assessed the timing of starting iron supplements, which ranged from 5 to 12 months. ^3^ Post hoc testing was performed using pairwise comparisons of the ranks; mean ranks within a row with different superscript letters were significantly different.

**Table 3:** Iron parameters at 12 months according to milk feeding type from 6 to 12 months.

	BF	FF	MF	*p* Value ^1^
	(*n* = 21)	(*n* = 62)	(*n* = 43)	
Hb (g/dL)	11.2 (10.9–12.4) ^a2^	12.6 (12.2–13.1) ^b^	12.2 (11.8–12.8) ^b^	<0.001
<10.5	4 (19.05)	2 (3.64)	1 (2.70)	0.037
≥10.5	17 (80.95)	53 (96.36)	36 (97.30)	
Hct (%)	38.6 (37.1–39.8)	39.4 (37.4–41.3)	38.8 (36.8–40.4)	0.162
<33	1 (4.76)	2 (3.64)	1 (2.70)	1.000
≥33	20 (95.24)	53 (96.36)	36 (97.30)	
MCV (fL)	83.0 (79.8–86.9)	83.6 (81.9–86.3)	83.5 (81.2–85.5)	0.364
<70	0	0	0	
≥70	21 (100)	54 (100)	37 (100)	
MCH (pg)	25.3 (24.4–26.4) ^a^	27.0 (26.0–27.6) ^b^	26.5 (25.7–27.2) ^b^	<0.001
<23	3 (14.29)	0	0	0.006
≥23	18 (85.71)	55 (100)	37 (100)	
MCHC (%)	30.5 (29.7–31.3) ^a^	32.0 (31.2–32.5) ^b^	31.8 (30.9–32.4) ^b^	<0.001
<30	10 (47.62)	2 (3.64)	2 (5.41)	<0.001
≥30	11 (52.38)	53 (96.36)	35 (94.59)	
RDW (%)	13.2 (12.5–15.0) ^b^	12.7 (12.1–13.2) ^a^	12.7 (12.1–13.2) ^a^	0.006
≥15	5 (25.00)	1 (1.89)	1 (2.86)	<0.001
<15	15 (75.00)	52 (98.11)	34 (97.14)	

Abbreviation; BF, breastfeeding; FF, formula feeding; MF, mixed feeding, Hb, hemoglobin; Hct, hematocrit; MCV, mean corpuscular volume; MCH, mean corpuscular hemoglobin; MCHC, mean corpuscular hemoglobin concentration; RDW, red cell distribution width. Values are presented as mean ± SE or median (IQR). The cutoff values for iron deficiency parameters are based on Korean age-specific reference ranges from Reference [22]. ^1^ *p*-values obtained by rank-based analysis of covariance (rank-based ANCOVA) for continuous variables after adjusting for sex, birth weight, and premature infant, and Fisher’s exact test for categorical variables. ^2^ Post hoc testing was performed using pairwise comparisons of the ranks; mean ranks within a row with different superscript letters were significantly different.

**Table 4:** Anthropometric and iron parameters at 36 months according to milk feeding type from 6 to 12 months.

	BF	FF	MF	*p* Value ^1^
	(*n* = 13)	(*n* = 35)	(*n* = 23)	
Height (cm)	93.70 (92.70–95.80)	96.00 (94.10–98.85)	95.60 (93.30–98.50)	0.189
Height-for-age Z-score	−0.37 (−0.89–−0.08)	−0.02 (−0.54–0.95)	−0.13 (−0.70–0.65)	0.144
Weight (kg)	13.70 (13.15–14.13)	14.70 (13.65–16.10)	14.40 (13.65–15.20)	0.171
Weight-for-age Z-score	−0.21 (−0.56–0.15)	0.36 (−0.13–0.95)	0.26 (−0.32–0.57)	0.162
Hb (g/dL)	12.30 (11.80–12.60)	12.80 (12.00–13.10)	12.30 (12.10–12.90)	0.618
Hct (%)	38.80 (37.50–39.30)	38.30 (37.00–40.20)	38.90 (36.55–40.95)	0.930
MCV (fL)	86.30 (85.20–88.70)	83.70 (82.10–86.40)	83.20 (81.35–85.85)	0.272
MCH (pg)	27.60 (26.90–28.50)	27.20 (26.60–28.10)	27.15 (26.25–27.75)	0.860
MCHC (%)	31.90 (31.30–32.50)	32.60 (31.80–33.80)	32.25 (31.60–32.90)	0.305
RDW (%)	12.40 (12.10–12.60)	12.25 (12.00–12.50)	12.50 (11.90–12.90)	0.425

Abbreviation; BF, breastfeeding; FF, formula feeding; MF, mixed feeding, Hb, hemoglobin; Hct, hematocrit; MCV, mean corpuscular volume; MCH, mean corpuscular hemoglobin; MCHC, mean corpuscular hemoglobin concentration; RDW, red cell distribution width. Values are presented as median (IQR) after adjusting for sex, birth weight and premature infant. ^1^ *p*-values obtained by rank-based analysis of covariance (rank-based ANCOVA).

## Data Availability

The data supporting the findings of this study are available from the corresponding author upon reasonable request. The data are not publicly available due to privacy and ethical restrictions.

## Supplementary Materials

The following supporting information can be downloaded at: https://www.mdpi.com/article/10.3390/nu18132197/s1, Table S1: Pairwise feeding group comparisons for weight at each follow-up time point; Table S2: Pairwise feeding group comparisons for height at each follow-up time point; Table S3: Pairwise feeding group comparisons for head circumference at each follow-up time point.

## Abbreviations

The following abbreviations are used in this manuscript:

BF	Breastfeeding
FF	Formula Feeding
MF	Mixed Feeding
