# Supplementary material for: Feeding Type at 6–12 Months and Early-Life Iron-Related Hematologic Changes in Infants: A Longitudinal Birth Cohort Study
Source: Nutrients. 2026 Jul 6;18(13):2197. doi: 10.3390/nu18132197 (PMC13364211; doi:10.3390/nu18132197)
Supplement: Supplementary file 1 [file nutrients-18-02197-s001.zip › nutrients-4361382-supplementary.pdf]

Table S1. Pairwise feeding group comparisons for weight Z-score at each follow-up time point

Contrast	Estimate	SE	p value[1)]
At birth			
BF - FF	-0.20	0.16	0.221
BF - MF	-0.11	0.19	0.564
FF- MF	-0.08	0.21	0.685
At 2 months			
BF - FF	-0.23	0.17	0.192
BF- MF	-0.08	0.20	0.666
FF - MF	-0.14	0.21	0.509
At 6 months			
BF - FF	-0.51	0.18	0.004
BF - MF	-0.56	0.20	0.006
FF - MF	0.06	0.22	0.805
At 12 months			
BF - FF	-0.25	0.17	0.145
BF- MF	-0.64	0.23	0.006
FF - MF	0.39	0.23	0.090

Abbreviation; BF, breastfeeding; FF, formula feeding; MF, mixed feeding

Values are presented as Estimate and SE after adjusting for sex and prematurity.

[1)] The p-values by generalized estimating equations (GEE).

Table S2. Pairwise feeding group comparisons for height Z-score at each follow-up time point

Contrast	Estimate	SE	p value[1)]
At birth			
BF - FF	-0.04	0.31	0.897
BF - MF	0.02	0.26	0.941
FF- MF	-0.06	0.29	0.837
At 2 months			
BF - FF	0.08	0.26	0.765
BF- MF	-0.62	0.23	0.008
FF - MF	0.69	0.25	0.006
At 6 months			
BF - FF	-0.41	0.22	0.063
BF - MF	-0.89	0.26	0.001
FF - MF	0.48	0.27	0.076
At 12 months			
BF - FF	0.08	0.23	0.742
BF- MF	-0.76	0.23	0.001
FF - MF	0.83	0.25	0.001

Abbreviation; BF, breastfeeding; FF, formula feeding; MF, mixed feeding

Values are presented as Estimate and SE after adjusting for sex and prematurity.

[1)] The p-values by generalized estimating equations (GEE).

Table S3. Pairwise feeding group comparisons for head circumference Z-score (HCZ) at each follow-up time point

Contrast	Estimate	SE	p value[1)]
At birth			
BF - FF	-0.21	0.24	0.380
BF - MF	0.11	0.28	0.690
FF- MF	-0.32	0.25	0.204
At 2 months			
BF - FF	-0.15	0.23	0.507
BF - MF	0.02	0.29	0.940
FF- MF	-0.17	0.31	0.569
At 6 months			
BF - FF	-0.15	0.24	0.521
BF - MF	0.18	0.28	0.514
FF- MF	-0.33	0.28	0.242
At 12 months			
BF - FF	-0.25	0.25	0.319
BF - MF	-0.14	0.34	0.690
FF- MF	-0.11	0.35	0.750

Abbreviation; BF, breastfeeding; FF, formula feeding; MF, mixed feeding; HCZ, head circumference-for-age Z-score

Values are presented as Estimate and SE after adjusting for sex and prematurity.

[1)] The p-values by generalized estimating equations (GEE).
